# A panel of genes methylated with high frequency in colorectal cancer

**DOI:** 10.1186/1471-2407-14-54

**Published:** 2014-01-31

**Authors:** Susan M Mitchell, Jason P Ross, Horace R Drew, Thu Ho, Glenn S Brown, Neil FW Saunders, Konsta R Duesing, Michael J Buckley, Rob Dunne, Iain Beetson, Keith N Rand, Aidan McEvoy, Melissa L Thomas, Rohan T Baker, David A Wattchow, Graeme P Young, Trevor J Lockett, Susanne K Pedersen, Lawrence C LaPointe, Peter L Molloy

**Affiliations:** 1 CSIRO Animal, Food & Health Sciences, Preventative Health Flagship, North Ryde, NSW, Australia; 2 CSIRO Computational Informatics, Preventative Health Flagship, North Ryde, NSW, Australia; 3 Clinical Genomics Pty Ltd, North Ryde, NSW, Australia; 4 Flinders Centre for Innovation in Cancer, Flinders University (FMC), Adelaide, SA, Australia

**Keywords:** Colorectal cancer, DNA methylation, Biomarker

## Abstract

**Background:**

The development of colorectal cancer (CRC) is accompanied by extensive epigenetic changes, including frequent regional hypermethylation particularly of gene promoter regions. Specific genes, including *SEPT9*, *VIM1* and *TMEFF2* become methylated in a high fraction of cancers and diagnostic assays for detection of cancer-derived methylated DNA sequences in blood and/or fecal samples are being developed. There is considerable potential for the development of new DNA methylation biomarkers or panels to improve the sensitivity and specificity of current cancer detection tests.

**Methods:**

Combined epigenomic methods – activation of gene expression in CRC cell lines following DNA demethylating treatment, and two novel methods of genome-wide methylation assessment – were used to identify candidate genes methylated in a high fraction of CRCs. Multiplexed amplicon sequencing of PCR products from bisulfite-treated DNA of matched CRC and non-neoplastic tissue as well as healthy donor peripheral blood was performed using Roche 454 sequencing. Levels of DNA methylation in colorectal tissues and blood were determined by quantitative methylation specific PCR (qMSP).

**Results:**

Combined analyses identified 42 candidate genes for evaluation as DNA methylation biomarkers. DNA methylation profiles of 24 of these genes were characterised by multiplexed bisulfite-sequencing in ten matched tumor/normal tissue samples; differential methylation in CRC was confirmed for 23 of these genes. qMSP assays were developed for 32 genes, including 15 of the sequenced genes, and used to quantify methylation in tumor, adenoma and non-neoplastic colorectal tissue and from healthy donor peripheral blood. 24 of the 32 genes were methylated in >50% of neoplastic samples, including 11 genes that were methylated in 80% or more CRCs and a similar fraction of adenomas.

**Conclusions:**

This study has characterised a panel of 23 genes that show elevated DNA methylation in >50% of CRC tissue relative to non-neoplastic tissue. Six of these genes (*SOX21*, *SLC6A15*, *NPY*, *GRASP*, *ST8SIA1* and *ZSCAN18)* show very low methylation in non-neoplastic colorectal tissue and are candidate biomarkers for stool-based assays, while 11 genes (*BCAT1*, *COL4A2*, *DLX5*, *FGF5*, *FOXF1*, *FOXI2*, *GRASP*, *IKZF1*, *IRF4*, *SDC2* and *SOX21*) have very low methylation in peripheral blood DNA and are suitable for further evaluation as blood-based diagnostic markers.

## Background

It is now well established that widespread epigenetic changes, including of DNA methylation profiles, relative to non-neoplastic tissue are a characteristic of many cancer types [1,2]. These changes typically involve the hypermethylation of promoter regions, characterised by CpG islands, of many genes as well as reduced methylation of repeated DNA sequences and some individual genes [2-4]. Hypomethylation of repeat sequences has also been associated with illegitimate recombination and chromosomal instability [5]. A wide range and number of genes are commonly methylated in different cancers, including colorectal cancer [4,6,7]. Promoter hypermethylation frequently occurs on genes that are already silent in non-neoplastic tissue [7,8], but is also associated with silencing of gene expression including that of tumour suppressor genes, such as *RB1*, *APC*, and other genes involved in cancer development, e.g. the *MLH1* DNA mismatch repair gene [3,4]. In addition to identifying genes with a potential role in oncogenesis, methylation of specific gene promoters can be a hallmark of different cancer types and can be used in diagnosis and classification of cancers [4]. In colorectal cancer, for example, co-ordinate methylation of a set of genes classifies cancers as CpG Island Methylator Phenotype (CIMP) and this classification is associated with mutations in the *BRAF* gene [9,10]. In an overlapping classification, approximately 20% of CRC has *MLH1* DNA mismatch repair gene promoter methylation and in turn, this methylation is associated with sporadic microsatellite-unstable CRC [11]. While many genes are relevant to CRC subtypes, some genes such as *SEPT9*[12] and *VIM*[13] become methylated in a high fraction of cancers and are being commercialised as diagnostic markers. Despite their promise, there is considerable potential for the development of new DNA methylation biomarkers or panels to improve the sensitivity and specificity of current cancer detection tests.

While promoter methylation was initially identified through individual candidate gene analyses, genome-wide techniques have rapidly broadened our understanding of the scope of DNA methylation changes. An early epigenome technique was the use of expression microarrays to examine expression reactivation after the application of a DNA methylation inhibitor, such as 5-aza 2′deoxycytidine (d-Aza), to a cancer cell line. As promoter methylation is commonly associated with gene silencing, a reactivation of gene expression serves as a proxy indicator of genes whose activity was silenced by such methylation. More recent advances in microarray technologies, particularly the Illumina Infinium 27 K and 450 K Bead Chip arrays [14], allow direct interrogation of DNA methylation in clinical samples at a large number of CpG sites. In addition, high throughput sequencing allows an even larger fraction of the methylome to be observed.

In this study, we have combined analysis of gene expression in colorectal cancer samples together with data from two new methods of genome-wide DNA methylation analysis that interrogate different subsets of CpG sites, Bisulfite-Tag [15] and a biotin-capture method Streptavidin bisulfite ligand methylation enrichment (SuBLiME) [16], in order to discover biomarkers. This approach has enabled us to identify a panel of genes that become methylated in a high proportion of colorectal cancers. Candidate biomarkers have been further evaluated and validated in colorectal tissues by multiplexed bisulfite sequencing and by quantitative methylation specific PCR on additional patient samples. We have further compared our candidates with previously published markers, including those identified in a number of recently published studies that used a variety of different genome-wide methods [6,7,17-27] and with data from The Cancer Genome Atlas consortium. Based on our analyses of tissues and comparison with publically available data, we have validated a panel of targets that become methylated at early stages of oncogenesis, for clinical evaluation as diagnostic biomarkers. The genes identified include both novel genes and genes previously identified in other studies.

## Methods

### Tissue specimens, cell lines and nucleic acids

DNA samples for Bisulfite-Tag genome-wide analysis, multiplexed bisulfite sequencing of amplicons, and methylation specific PCR (MSP) assays were drawn from the sample collection below. Colorectal tissue specimens obtained from surgical resections were fresh-frozen and stored at -80°C. Access to the tissue bank for this research was approved by the Research and Ethics Committee of the Repatriation General Hospital and the Ethics Committee of Flinders Medical Centre, both in Adelaide, South Australia. Colorectal tissue specimens were classified as non-neoplastic (59), adenoma (13) or adenocarcinoma (95 comprising 24 Dukes A, 18 Dukes B 45 Dukes C and 8 Dukes D) on the basis of histological assessment by an expert pathologist. An additional panel of cancer tissue (10), matched non-neoplastic tissue (10) and adenoma tissue (10) samples was purchased from Bioserve Biotechnologies (Beltsville, MD).

Culture conditions for the colorectal cancer (CRC) cell lines HCT116, HT29, SW480and LIM1215 and treatment with 5-aza 2′deoxycytidine (d-Aza) and trichostatin (TSA) are described in Additional file 1. RNA was isolated using Promega SV total RNA purification kits.

DNA was isolated from frozen tissue samples (20 mg each) following homogenisation using a Retsch TissueLyser (Qiagen) in the presence of 600 μL of chilled Nucleic Acid Solution (Promega Wizard DNA Purification kit). DNA was then isolated following the recommended protocol of the kit. DNA fully methylated at CpG sites, CpGenome DNA, was purchased from Millipore (Cat No. S 7821). DNA from pooled peripheral blood of healthy individuals (wbc DNA) was purchased from Roche Applied Science (Cat No. 05619211001).

### Gene expression arrays

Levels of gene expression in CRC cell lines with or without d-Aza and/or TSA treatment were determined using Affymetrix Exon 1.0ST gene chips. cDNA was prepared and labelled using the High Capacity cDNA Reverse Transcription Kit from Applied Biosystems (Part No. 4368814) and gene chip hybridisation and washing done according to Affymetrix protocols detailed in the GeneChip® Whole Transcript (WT) Sense Target Labeling Assay Manual P/N 701880 Rev. 4. Microarrays were processed and analysed using R/Bioconductor. Arrays were normalized using robust multiarray normalization (RMA), implemented in the *simpleaffy* package [28]. Probesets with differential expression (treated vs control) within cell lines were identified using *limma*[29].

### Genome-wide DNA methylation analysis

Genome-wide DNA methylation analysis using SuBLiME has been described previously [16]. Libraries of SuBLiME-captured DNA from three cell lines and from wbc DNA were sequenced using ABI SOLiD 3 chemistry and reads aligned to the genome [16]. Cytosines in these fragments were counted and the summed counts across reads used to identify sites that showed statistically significant (*p* < 0.01) elevated methylation, as determined by the *edgeR* R/Bioconductor package [30]. Bisulfite-Tag measures methylation at TaqI (5′-T^CGA) and MspI (5′-C^CGG) sites across the genome [15]. Briefly, the method relies on cutting of genomic DNA with TaqI and MspI, enzymes that both cut DNA independently of methylation at the central CG of their recognition sites. Following restriction enzyme digestion the DNA was treated with sodium bisulfite without denaturing the double-stranded fragments. Thus only the two base overhang reacts with bisulfite, with unmethylated cytosines being converted to uracils and methylated cytosines remaining unconverted. Separate linkers with appropriate matching overhangs were ligated to the bisulfite converted ends, allowing separate amplification of populations representing methylated and unmethylated DNA. Following labelling with either Cy3 or Cy5 dyes, methylated and unmethylated fractions were hybridized with Nimblegen 720 K Promoter tiling arrays [15]. Arrays were scanned using the Axon GenePix 4000b and the Perkin Elmer ScanRI and methylation at individual TaqI or MspI sites determined as described in Additional file 1.

### Multiplex bisulfite sequencing

DNA (3 to 7 μg) extracted from 10 colorectal and 10 matched non-neoplastic tissue specimens (Flinders Medical Centre, above) was bisulfite converted using the EZ Methylation-Gold kit (Zymo Research) as recommended by the manufacturer, except for using the following modification to the bisulfite reaction temperature conditions: 99°C for 5 min, 60°C for 25 min, 99°C for 5 min, 60°C for 85 min, 99°C for 5 min and 60°C for 175 min. The concentration of purified bisulfite-converted DNA was determined by quantitative real-time PCR using bisulfite conversion-specific primers for *ACTB*[12].

A total of 59 conversion specific PCRs across 27 genes in triplicate (primers and PCR conditions described in Additional file 1 and Additional file 2: Table S3) were applied to 5-10 ng bisulfite treated DNA including, peripheral blood lymphocyte DNA (wbc DNA, Roche Applied Science, Cat # 1 691 112) and a 1:1 mix of wbc DNA and enzymatically methylated DNA (CpGenome™ Methylated DNA, Millipore). The triplicates were pooled and the concentration of PCR products estimated by gel electrophoresis.

Equivalent amounts of the above 59 amplicons (approximately 15-20 ng) derived from the same patient or control samples were pooled, resulting in 22 pools. A total of 500 ng of each DNA pool was ligated with a bar-coded “MID” linker (Roche Applied Science) so that the sample of origin for each read could be deduced from the sequence. Libraries of pooled amplicons were prepared following protocols provided with the Roche Library Preparation Kit and reagents, except that Qiagen MinElute columns were used to remove excess MID linkers. The libraries were sequenced on two halves of a flow cell on the Roche 454 Titanium FLX system; one half contained all of the CRC samples and the other half the equivalently bar-coded normal samples. Bisulfite sequencing reads were assigned to individual tissue samples using the bar-code MID sequences and aligned against *in silico* bisulfite-converted reference sequence with all ‘C’ characters at CpG sites converted to ‘Y’ and ‘C’ in all other contexts converted to ‘T’. After best alignment with SHRiMP V2.04 [31], the fraction of unconverted cytosines at each potential CpG methylation site was determined for each sample. Samples from wbc DNA as well as a 1:1 mixture of methylated (CpGenome™) and wbc DNAs were analysed for quality control purposes.

### Quantitative assays for DNA methylation

Methylation specific PCR assays and control “cytosine free fragment” (CFF) assay [12] were performed using primer pairs and assay conditions shown in Additional file 2: Table S4. Input levels of bisulfite-treated DNA were quantified using by qPCR using the CFF assay and a standard curve of serially diluted human genomic DNA (Roche Applied Science) ranging from 100 ng to 100 pg. For each target fragment, amounts of methylated target DNA were quantified using a standard curve of fully methylated DNA, 40 pg, 200 pg, 1 ng and 5 ng (CpGenome™ DNA, Millipore,) mixed with unmethylated DNA (Roche Applied Science) to give a total of 5 ng DNA. The levels of methylated DNA of each sample were determined from the standard curve and combined with the amount input DNA to calculate the percentage methylation.

## Results

### Biomarker discovery strategy

**Figure 1:**
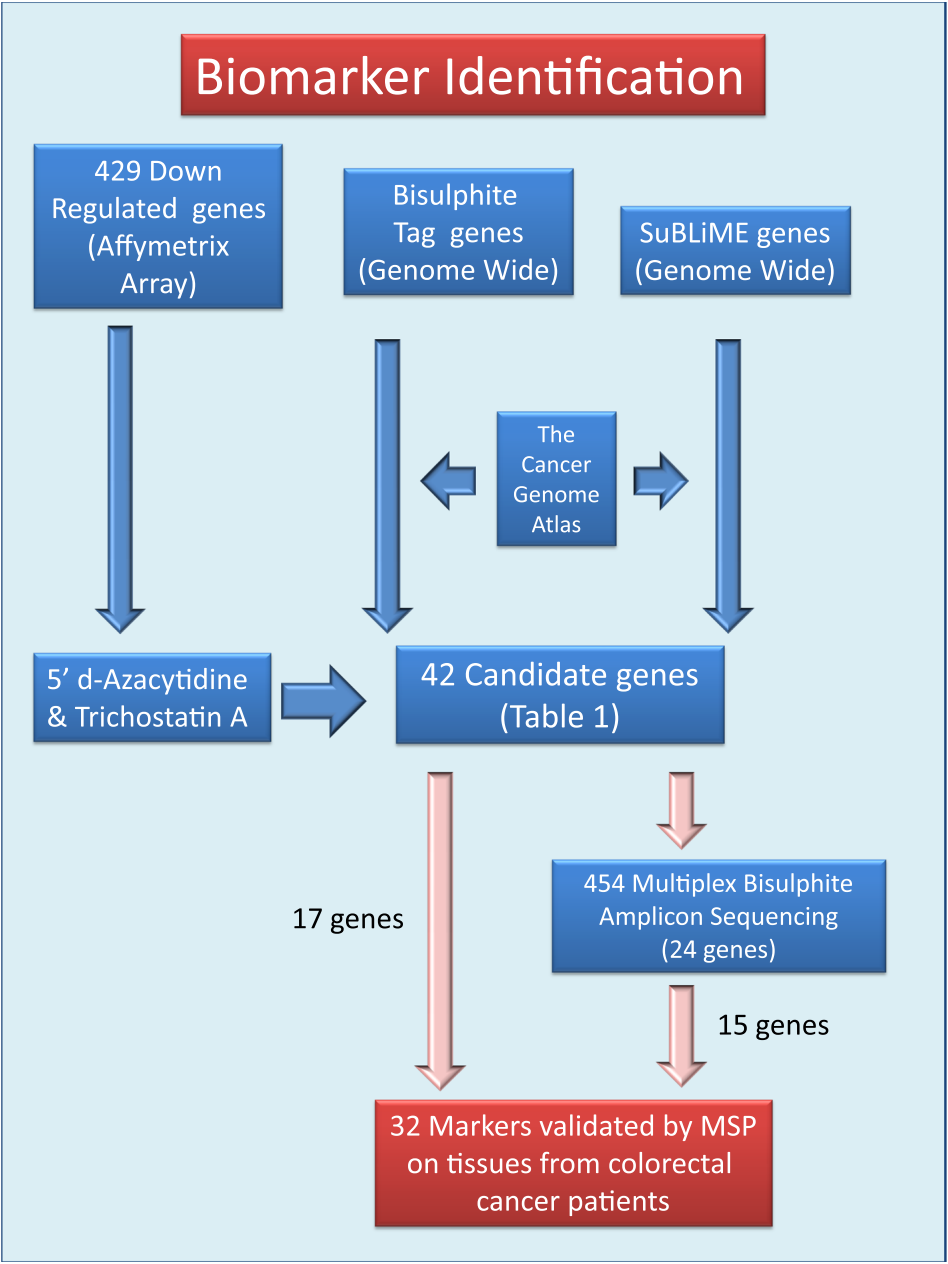
**Biomarker discovery scheme.** Detail discussed in text.

In order to identify DNA methylation biomarkers potentially suitable for early diagnosis of colorectal cancer, we have combined different genome-wide approaches as illustrated in Figure 1.

• We had previously identified [32] in a large set of colorectal tissues a panel of 429 genes that were down-regulated in both colorectal cancers and adenomas relative to normal tissue. Our initial approach for identification of potential DNA methylation biomarkers focused on this panel of genes. We used activation of gene expression in cell lines, following treatment with d-Aza alone or in combination with TSA as a first approach (Figure 1, left arrows).

• In parallel, we had developed two novel methods of genome-wide DNA methylation analysis, Bisulfite-Tag and SuBLiME, that interrogated different but overlapping portions of the methylome (Figure 1); these were applied to clinical specimens and/or CRC cell lines and wbcDNA respectively. Initially, the genome-wide methylation data was specifically examined for evidence of enhanced methylation among the 429 panel of down-regulated genes (Table 1).

• Genome-wide analysis of the Bisulfite-Tag data also identified a novel set of genes that showed differential methylation between CRC and matched non-neoplastic tissue DNAs.

• Likewise, analysis of SuBLiME data on methylation in three CRC cell lines compared with wbc DNA from normal subjects identified a further panel of candidate biomarkers. This panel was further filtered to select genes for which there was evidence of differential methylation in clinical specimens – initially in Bisulfite-Tag data and subsequently in 27 K Infinium BeadChip array data from The Cancer Genome Atlas (TCGA) consortium when that became publically available.

**Table 1:** Summary of genes and analyses

	**A**	**B**	**C**	**D**	**E**	**F**	**G**	**H**	**I**	**J**
**Gene**	**Down-regulated**[32]	**d-Aza/TSA activation**	**Bis-Tag tissue**	**Bis-Tag cells**	**SuBLiME**	**SuBLiME rank**[16]	**TCGA**	**Literature**	**Roche 454 sequencing**	**Tissue qMSP**
ADAMTS1	Y	N	10	2	2	#	Y	[7,21,22,24]	Y	
ANK2	Y	Y					Y			Y
CA4	Y	Y					Y	[7]		Y
CFD	Y	N								Y
CHRDL1	Y	Y					Y	[7]		Y
COL1A2	Y	Y	10	2	4	699	Y	[7]	Y	
COL4A1	Y	Y			2	1385	Y	[7,24]	Y	Y
COL4A2	Y	Y	2	2	2	1608	Y	[7,21]	Y	Y
CXCL12	Y	Y					Y	[7]		Y
EDIL3	Y	Y	10	2	4	#	Y	[7,21]	Y	Y
EFEMP1	Y	Y			4	122	Y	[7,18,21]	Y	Y
EPB41L3	Y	Y			2	1217		[7]		Y
FBN1	Y	Y	10	2	4	705	Y	[7,24,26]	Y	
FGFR2	Y	(+/-)	10	2	4	#	Y		Y	
FOXF1	Y	N	10	2	4	82	Y	[7]	Y	Y
MAFB	Y	(+/-)	8	2	2	835	Y	[7,24]		Y
MAMDC2	Y	Y					Y			Y
MEIS1	Y	Y	10	0	4	#	Y	[7]	Y	
MMP2	Y	Y	10	4	2	446	Y	[7,17,18]	Y	
MT1M	Y	N						[7]		Y
PPP1R14A	Y	Y			4	320	Y	[7,21,23]	Y	
SCNN1B	Y	Y					Y	[18,24]		Y
SDC2	Y	Y	10	4	4	300	Y	[7,24]	Y	Y
TCF21	Y	Y	10	4	2	1420	Y	[7]	Y	
ZSCAN18 (ZNF447)	Y	Y			4	245		[18,20,24]		Y
BCAT1	-				Y	137	Y	[7,24]		Y
DLX5	-		Y		Y	249	Y	[23,24]	Y	Y
FGF5	-		Y					[7,17]	Y	Y
FOXB1	-		(Y)		Y	32	Y		Y	Y
FOXD2	-		Y				Y	[7,18]	Y	
FOXI2	-				Y	9	Y	[7]		Y
GRASP	-		(Y)		Y	76	Y	[7,24]	Y	Y
IKZF1	-				Y	1	N	[7,33]		Y
IRF4	-				Y	27	Y	[7,18,20,24]		Y
IRX1	-		Y		Y	47		[7]	Y	Y
NPY	-		(Y)		Y	36	Y	[7,17,18,24,27]	Y	Y
PDX1	-		Y				Y	[7]	Y	Y
SEPT9	-								Y	Y
SLC6A15	-		Y		Y	1136	Y	[7,18,20,24]		Y
SOX21	-		(Y)		Y	6	Y	[7]	Y	Y
ST8SIA1	-				Y	8	Y	[7]		Y
SUSD5	-		(Y)		Y	195		[7]	Y	
ZNF471	-		Y		Y	18	Y	[7]	Y	Y
**Controls**										
SEPT9	-								Y	Y
TMEFF2	-							[7,17,21,24,27]	Y	

Notes/Column.

A. Down-regulated: designated ‘Y’ if gene was in list of differentially-expressed (down-regulated) genes identified in LaPointe et al., 2012 [32].

B. D-AzaC/TSA activation; Genes with a 2-fold or greater change in gene expression ‘Y’, less than 2-fold ‘N’, borderline ‘(+/-)’.

C. Bis-tag tissue: for genes initially recognised as down-regulated (rows 3-27), genes were scored for methylation difference between cancer and normal tissues on a scale of 0 to 10.

For genes in rows 29-47, those designated ‘Y’ were among the top differentially methylated genes identified by Bis-tag (Additional file 2: Table S4). Those designated ‘(Y)’ were identified from SuBLiME data and differential methylation in clinical samples confirmed by inspection of Bis-tag plots.

D. Bis-tag cells: for genes initially recognised as down-regulated (rows 3-27), genes were scored for methylation in CRC cell lines on a scale of 0 to 4.

E. SuBLiME: for genes initially recognised as down-regulated (rows 3-27), genes were scored for methylation in CRC cell lines on a scale of 0 to 4. For genome-wide analysis (Rows 29-47), ‘Y’ indicates that gene was in list of differentially methylated genes (Ross et al. [16]).

F. SuBLiME rank: shows ranking within list of differentially methylated genes.

‘#’ differential sites (Column E) for these genes were either not found in two or more cell lines or were located in regions outside the promoter region (-2 kb to + 1 kb of UCSC canonical transcription start site) surveyed in Ross et al. [16].

G. TCGA: ‘Y’ denotes that differential methylation is confirmed in TCGA Illumina 27 K bead Chip data.

H. Literature: references demonstrating methylation of gene in colorectal cancer.

I. Roche 454 sequencing: ‘Y’ denotes included in multiplexed bisulfite sequencing.

J. MSP on Tissues: ‘Y’ denotes include in MSP quantification of methylation levels in CRC tissue sample.

From a combined analysis of our datasets (see Additional file 1, Section 4) we developed a prioritised list of genes for further evaluation by multiplexed bisulfite sequencing and methylation-specific PCR providing a detailed analysis of clinical samples.

### Genes down-regulated in colorectal cancer

We have previously identified in a large discovery set of colorectal tissues and in a separate validation set, a panel of genes that were down-regulated in colorectal neoplasia relative to non-neoplastic colon tissue [32]. Additional file 2: Table S1 provides an updated gene list for 429 genes down-regulated in neoplasia (adenoma and carcinoma combined, compared with non-neoplastic tissue) and 159 genes that are significantly down-regulated in adenomas. To further identify which of these might be down-regulated by DNA methylation we treated four colorectal cancer cell lines with d-Aza alone or in combination with TSA (Additional file 1). We identified treatment conditions that provided maximal DNA demethylation, as assessed by hypomethylation of Alu repeat sequences (Additional file 2: Table S2) and compared expression levels of treated and untreated cells using Affymetrix 1.0ST Exon arrays. We considered the set of 429 candidate down-regulated genes and assessed their level of activation in the different cell lines. Ratios of expression of treated compared with untreated samples were determined. For each candidate gene, ratios of expression of individual exonic probesets were determined and log_2_ transformed. Then for each cell line, the mean log_2_ fold-change across the four cell lines was used to rank genes; log_2_ fold-change data for genes that were analysed further are shown in Additional file 2: Table S2. It is notable that among the 20 genes scored as being activated, 17 have been shown in recent data sets to be commonly methylated in CRC, e.g. *EFEMP1*, *SDC2*, *EDIL3* (Table 1), while two (*ANK2* and *MAMDC2*) had not been reported to methylated in CRC. In recent TCGA consortium data [34] all but two of the 19 genes (*EPB4IL3* and *ZSCAN18*) show evidence of methylation in cancer.

### Genome-wide analysis of DNA methylation

We have used two novel methods of genome-wide DNA methylation analysis to directly identify genomic regions hypermethylated in CRC. The first of these methods, Bisulfite-Tag, analyses methylation at CpG sites contained with TaqI (5′-T^CGA) or MspI (5′-C^CGG) restriction enzyme sites. After digestion with these non-methylation-sensitive enzymes, the two base –CG overhangs are reacted with sodium bisulfite [15] such that unmethylated cytosines are converted to uracils, while methylated cytosines remain unreacted, (described in more detail in Additional file 1). This allows selective ligation of linkers to fragments methylated or unmethylated at the cut sites. The second method, SuBLiME, enriches for methylated DNA fragments in sodium bisulfite DNA by incorporation during primer elongation of biotin-14-dCTP at positions opposite 5′-methylcytosine. As the only remaining cytosines in bisulfite treated DNA are those sites methylated in the original DNA, the SuBLiME method specifically labels these sites for downstream purification of methylated fragments and subsequent deep sequencing. In this instance the DNA was also cut with Csp6I (5′-G^TAC) prior to enrichment to limit sequencing to the 50 bp around Csp6I cut sites.

As applied in this study, each method interrogated different, but overlapping, portions of the methylome. Notably both methods depend only on the methylation at single CpG sites for enrichment and so differ in coverage from methods that combine antibody or methylated DNA binding protein fractionation of the genome with microarray or sequence analysis, as these latter methods depend on the density of methylation. Likewise the novel methods employed here differ in coverage from other complexity-reduction methods such as RRBS [35] that tend to be biased toward CpG islands.

### Bisulfite-tagging

Methylated and unmethylated Bisulfite-Tag populations of DNA were amplified following fractionation from (1) eight individual CRC tissue samples and their matched non-neoplastic tissue, (2) pooled DNA of the eight cancers (3) pooled DNA from the eight matched normal tissues and (4) four CRC cell lines (HCT116, HT29, Caco2 and LIM1215). Methylated and unmethylated Bisulfite-Tag fractionated DNAs were hybridised to Nimblegen 720 K promoter tiling arrays. In the first instance we examined the methylation profile across genes that we had previously identified as down-regulated in CRC. Twelve of these genes were scored as methylated in CRC tissue samples or cell lines (e.g. *ADAMTS1*, *COL1A2*, *MAFB* and *SDC2*, Table 1). For genome-wide analysis, each sample had methylation scores at individual probes derived from the ratio of the methylated fraction signal over that of the unmethylated fraction signal and these scores were used to derive a metric of differential methylation between cancer and normal tissue by taking the difference between the scores for the cancer tissue and non-neoplastic tissue (Additional file 1). Since the number of assessable sites varied between genes and to minimise effects arising from single probes, scoring was based on differential methylation of either the top 2 or top 4 probes. Additional file 2: Table S5 provides a list of 41 genes ranked by fold-change showing the greatest differential methylation. Of these genes, three (*DLX5*, *FOXD2* and *SLC6A15*) have been reported by others to be methylated in CRC. Seven of these Top 41 genes plus a further 5 genes that were supported by both Bisulfite-tag and SuBLiME data were chosen for detailed bisulphite sequencing and/or qMSP analysis (Table 1); see Discussion below and in Additional file 1, Section 4.

### SuBLiME

SuBLiME [16], was used to identify CpG sites that were methylated in at least two of three CRC cell lines, SW480, HCT116 and HT29, but not methylated in pooled wbc DNA of normal individuals. We reasoned that for future use as biomarkers for detection of cancer-derived DNA in plasma or serum, it would be important to choose regions that showed minimal methylation in blood of individuals without CRC. In the present application we used a reduced-representation version of SuBLiME in which all fragments were adjacent to Csp6I (5′-G^TAC) restriction sites. The reduced representation introduced by cutting the DNA with Csp6I introduces an arbitrary patchiness to the methylome information. To direct biomarker discovery towards certain genes, differentially methylated CpG sites (DMC) proximal to gene transcription site starts (2 kb upstream to 1 kb downstream) were grouped. From this grouping, 1769 genes were identified as having promoter proximal DMC in at least two of the three pairwise comparisons to peripheral blood DNA [16]. Genes were ranked by the average number of DMC across the comparisons. This “weight-of-evidence” ranking approach biases toward gene loci hypermethylated in all three cell lines but not in blood and towards genes having CpG-rich regions around a number of Csp6I cut sites. The rank order of a gene within this list is shown in Table 1. Additional file 2: Table S6 provides a list of differentially methylated genes.

Since this dataset was developed using CRC cell lines, we first compared SuBLiME data with Bisulfite-Tag data from clinical samples. Though each method interrogates a different fraction of CpG sites and cell lines compared with tumours, 16 of the top 38 genes selected by Bisulfite-Tag were also identified among those genes showing significantly differential methylation between CRC cell line DNA and wbc DNA in the SuBLiME data (Additional file 2: Tables S5 and S6); this included two genes, *IRX1* and *ZNF471* ranked within the top 50 by both methods. In addition, we also examined, where possible, Bisulfite-Tag methylation profiles of genes identified as most differentially methylated in the SuBLiME analysis in order to confirm differential methylation in clinical samples. Five highly-ranked genes in the SuBLiME data, *GRASP*, *FOXBI*, *NPY*, *SOX21* and *SUSD5* were identified as showing evidence of differential methylation in Bisulfite-Tag methylation profiles (Table 1).

### Selection of genes for further analysis

To provide an initial priority list of genes for more detailed study we combined evidence from the different experimental data (see also Additional file 1, Section 4). We first scored within the candidate list of genes down-regulated in CRC as this list derived from a large clinical discovery data set and subsequent validation data set. The top half of Table 1 contains genes from this dataset. Based on a combined scoring of gene activation in response to d-Aza/TSA treatment, evidence of methylation in Bisulfite-Tag data (Additional file 2: Table S5) as well as existing literature data (*ADAMTS1*, *COL4A1*/*2*, *EFEMP1* and *PPP1R14A*, Table 1), fourteen genes were selected for bisulfite sequencing analysis.

We further included 11 (*DLX5*, *FGF5*, *FOXB1*, *FOXD2*, *GRASP*, *IRX1*, *NPY*, *PDX1*, *SOX21*, *SUSD5* and *ZNF471*) genes derived solely from DNA methylome analysis. These comprised top ranking genes arising from Bisulfite-Tag analysis of clinical samples (Additional file 2: Table S5) and those from SuBLiME analysis of CRC cell lines (Additional file 2: Table S6) that also showed evidence of methylation in the clinical sample Bisulfite-Tag data (Table 1).

Subsequently, as Infinium HumanMethylation 27 K BeadChip methylation data produced by The Cancer Genome Atlas Consortium [34] became available, we reanalysed the raw data using the R ‘lumi’ package [36] to preprocess and the ‘limma’ package [29] to discover differential methylation. A linear model incorporating disease state (165 CRC tumours versus 37 non-neoplastic colon tissue) with patient gender as a covariate was used in the analysis. These data were used to complement our approaches and to identify additional genes; especially from the SuBLiME data, for which there was clear evidence of methylation in a high fraction of TCGA clinical samples (Table 1, *BCAT1*, *FOXI2*, *IKZF1*, *IRF4*, *SLC6A15*, and *ST8SIA1*). These six newly identified genes formed part of the set of genes for which MSP assays were used to quantify levels of methylation in additional CRC samples. Plots of methylation in TCGA data at promoter probes of 15 genes that we had identified as differentially methylated in our Bisulfite-Tag or SuBLiME data are shown in Figure S1 (Additional file 1). With the exception of *IKZF1*, where probes are not located in the same region as identified by us, one or both interrogated probes show clear differential methylation.

### Deep bisulfite -sequence analysis of candidate genes

For the 25 genes chosen above we designed 1 to 5 pairs of primers for amplification from bisulfite-treated DNA of sequences in or around their promoters. A total of 59 amplicons, including for the control *SEPT9* and *TMEFF2* genes, were prepared from DNA of each of 10 CRC and matched non-neoplastic tissues, as well as controls of pooled wbc DNA from individuals without cancer, fully methylated DNA (CpGenome™) and a 50:50 mix of wbc and fully methylated DNAs. Barcoded linkers were separately ligated to pools of amplicons from each DNA source and multiplexed samples were sequenced on a Roche 454 GS FLX Titanium sequencer.

**Figure 2:**
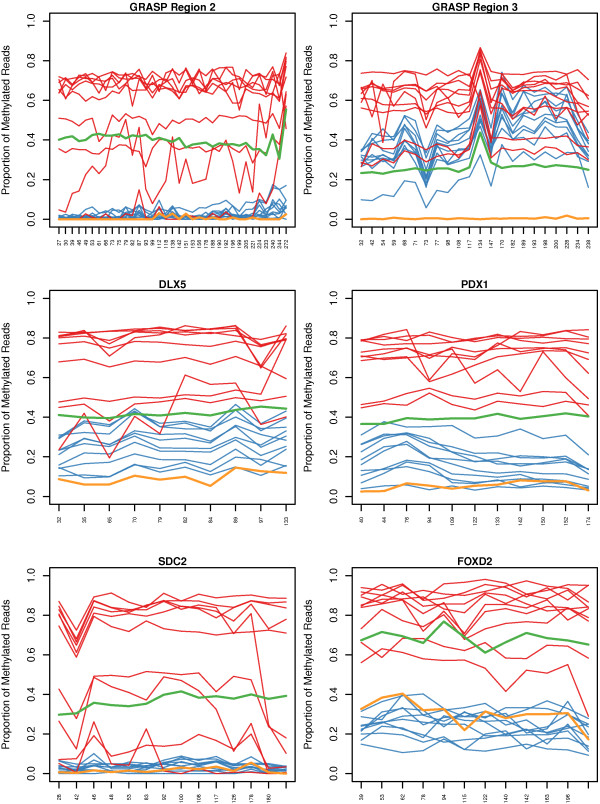
**Profiles of gene methylation for six amplicons.** Individual panels show plots of CpG site methylation across the indicated amplicons. Data is presented for 10 individual cancer tissues (red), 10 matched non-neoplastic colon tissues (blue), a 50:50 mix of wbc DNA and fully methylated DNA (green) and wbc blood DNA (ochre). CpG sites are equispaced along the x-axis with labels showing the relative position of each CpG site within the amplicon, relative to the start of the forward primer. Chromosomal locations of amplicons are provided in Additional file 2: Table S3. The y-axis shows the proportion of methylated cytosines at a CpG site. Sudden coordinated changes in measured methylation rate, such as that at coordinate 134 of the *GRASP* Region 3 amplicon, is due to a DNA alignment technical artefact caused by long thymine homopolymer repeats creating errors within the pyrosequencing reads.

Methylation profiles across individual amplicons are shown in Figure 2. The data for 59 amplicons representing 27 genes or regions (Additional file 2: Table S3) is summarised in Additional file 2: Table S7. The table shows the approximate range of methylation levels at CpG sites across each amplicon for the individual cancer samples. For the ten patients, the number showing high level (>50%) or partial (20 to 50%) methylation is shown in Additional file 2: Table S7, columns C and D respectively, for each amplicon. Methylation of three of these genes, *SEPT9*, *TMEFF2* and *ADAMTS1*[22,37,38] has been previously reported in colorectal cancer and they show partial or high level methylation in 10, 10 and 7 cancer DNAs, respectively. Among the 24 additional genes tested, the *FGFR2* gene showed only marginally significant differential methylation between cancer and matched non-neoplastic tissue (Additional file 2: Table S7). Notably the region initially identified from SuBLiME data and targeted for sequencing lies about 2 kb downstream of the transcription start site. Most genes showed differential methylation in a high proportion of samples. In summary, 9 genes - *DLX5*, *FOXD2*, *IRX1*, *MEIS1*, *MMP2*, *NPY*, *PDX1*, *SUSD5* and *TCF21*- showed high or partial methylation in all 10 samples, 9 genes - *COL1A2*, *COL4A*, *EFEMP*, *FGF5*, *FOXF1*, *GRASP*, *SDC2*, *SOX21* and *ZNF471* – in 9 samples, *FOXB1* in 8 samples, *PPP1R14A* in seven, *FBN1* and *EDIL3* in six and *MEIS1* in three samples. In some cases, e.g. *EDIL3*, *FBN1*, *GRASP* (Region 2), *MEIS1* and *SDC2*, the level of methylation in matched non-neoplastic colonic tissue was consistently very low. For other genes or regions, e.g. *DLX5*, *GRASP* Region 3, *IRX1*, *MMP2*, *NPY*, *PDX1* and *TCF21*, significant levels of methylation were evident in the matched normal tissue but methylation was always significantly increased in the cancer tissue. The data also demonstrates that for a given gene, not all regions show equivalent cancer-specific methylation. For example, for the *COL4A* gene(s) Regions 1 and 5 show high or partial methylation in 9 of 10 cancer samples, while Regions 2 and 3 are methylated in only 4 or 2 samples, respectively. *COL4A* Region 1 lies within the *COL4A1* gene, while *COL4A* Region 5 lies within the neighbouring, divergently transcribed *COL4A2* gene.

The sequencing data thus demonstrates colorectal cancer-specific DNA methylation for regions of 23 genes (*COL1A2*, *COL4A1*, *COL4A2*, *DLX5*, *EDIL3*, *EFEMP*, *FBN1*, *FGF5*, *FOXB1*, *FOXD2*, *FOXF1*, *GRASP*, *IRX1*, *MEIS1*, *MMP2*, *NPY*, *PDX1*, *PPP1R14A*, *SDC2*, *SOX21*, *SUSD5*, *TCF21* and *ZNF471*) and specific regions that may be used for development of assays to distinguish cancer from normal DNA.

### Methylation specific PCR assessment of methylation in colorectal tissue samples

**Figure 3:**
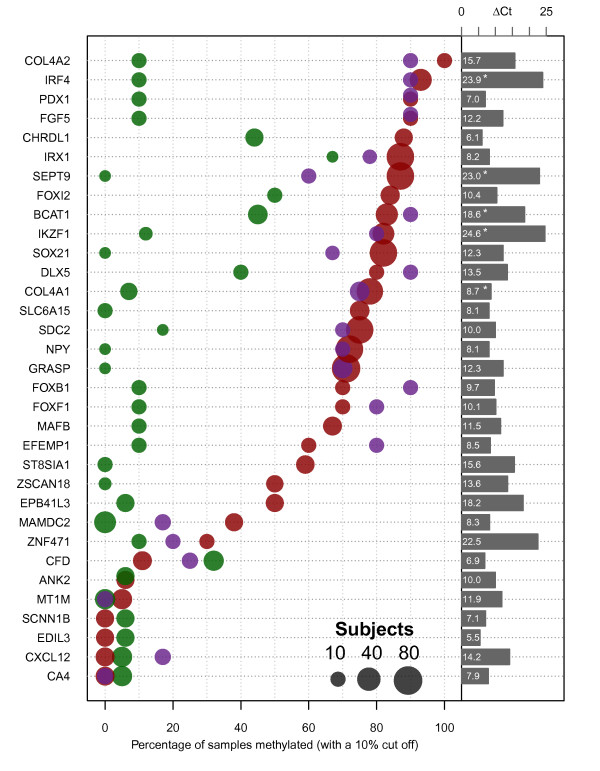
**Frequency of gene methylation in colorectal neoplasia.** Methylation levels of individual genes (left hand labels) were determined by qMSP using primer pairs and conditions described in Additional file 2: Table S4. The percentage of samples showing greater than 10% methylation is shown for CRC (red spots), matched normal tissue (green) and adenomas (purple). Up to 78 cancer samples were tested for any individual gene. The size of the spots is proportional to a log2 transformation of the number of samples tested (small gray circle10; medium gray circle 40; large gray circle 80). The difference in detection cycle between CpGenome™ DNA and wbc DNA (ΔCt ) is presented as bars to the right with lengths proportional to the ΔCt value (which is also presented numerically within each bar). An asterix denotes the qMSP reaction completed before reaction products from wbc DNA were detected, so the ΔCt is at least this value.

To further prioritise genes, MSP assays were designed for 32 of the list of 42 candidate genes in Table 1 and used to quantify levels of methylation in additional cancer, adenoma and non-neoplastic colon tissue samples (Figure 3 and Additional file 2: Table S8). Numbers of samples assessed for each gene are given in (Additional file 2: Table S8) and details of primers and assay conditions in (Additional file 2: Table S4). The choice of primer positions was guided by bisulfite sequencing data and/or sites showing differential methylation in SuBLiME, Bisulfite-Tag or TCGA Infinium HumanMethylation 27 K array data. The genes *ANK2*, *CA4*, *CFD*, *CHRDL1*, *CXCL12*, *MAMDC2*, *MT1M* and *SCNN1B* were selected directly from the original list of genes down-regulated in CRC [32]. Among these genes, only *MAMDC2* and *CHRDL1* showed methylation in a significant fraction of CRC samples. For the remainder of the genes, their selection had been based on input from genome wide analyses and, as expected, frequent methylation was evident in both CRC and adenomas. Eleven genes were methylated in 80% or more of the tested cancers, with six showing equal or greater frequency of methylation than the *SEPT9* marker (Figure 3). Notably, a number of genes also showed a higher frequency of methylation in adenomas. Of the eleven genes, only SOX21 was unmethylated in all matched normal tissues tested.

To inspect correlations between markers and individual tumors we ordered the qMSP results using hierarchical clustering and created a heatmap to identify the subsets of tumours and their corresponding methylated markers (Additional file 1: Figure S2). For a closer examination of co-methylation between individual pairs of qMSP biomarkers we created a pairs plot (Additional file 3: Figure S3). This presentation of the data allows identification of pairs of markers that are highly concordant or discordant in methylation levels across the tumors, aiding the grouping of markers into panels for greater biomarker sensitivity.

To construct the heatmap, it was necessary to exclude 34 tumors with incomplete marker information. The heat map incorporates two sets of data; qMSP results for seven markers across 75 tumors and an expanded set of 12 markers across a further 20 tumors. The pairs plot shows that methylation of some genes is highly correlated, e.g. *IRF4*, *BCAT1*, *FOXI2*, while that of *SEPT9* is most closely correlated with *GRASP*, *SDC2* and *SOX21*. This could reflect underlying co-ordinate methylation or high level methylation of these genes within cancer cells combined with different proportions of cancer cells within the tissue samples. In contrast, other gene pairs, e.g. *GRASP* and *NPY,* or *IRX1* and *GRASP,* or *IRX1* and *SDC2*, are commonly methylated but show little correlation in measured levels of methylation within individual cancer samples (Additional file 3: Figure S3).

Both the frequency (proportion of tumors) and extent (level of methylation within a tumor) of gene methylation should predict the ability to detect specific methylated DNA sequences derived a tumor in either blood or feces. Although the numbers of comparisons are limited, Inspection of (Additional file 3: Figure S3) shows that the relative levels of individual gene methylation vary significantly between tumors and suggests that certain combinations of genes could provide for increased sensitivity of cancer detection.

### Methylation levels in wbc DNA

Another important factor in identification of candidates for further development as blood-based biomarkers for cancer diagnosis is the potential for background levels of methylated sequences in plasma of healthy subjects to lead to false positive tests [19]. The most likely source of DNA in plasma is through release from white blood cells in vivo or through cell lysis during blood handling and plasma isolation. We applied the MSP assays used for tissue analysis to pooled wbc DNA from normal individuals (Roche) and compared amplification with that from fully methylated DNA (CpGenome™). The delay in amplification of methylated sequences from wbc DNA compared with that from fully methylated DNA provides a measure of the level of methylation in wbc DNA (Figure 3). Eleven of the genes showing 70% or greater frequency of methylation in cancers and/or adenomas and also showed less than an estimated 0.1% rate of methylation in wbc DNA (considering a cut-off of 10 PCR cycles between fully methylated and wbc DNA). Combined, the frequent methylation in CRC and the very low background of methylated DNA seen in wbc DNA suggests that *IRF4*, *BCAT1* and *IKZF1*, similarly to *SEPT9*, are excellent candidate biomarkers, while additional genes such as *COL4A2*, *SOX21*, *DLX5* and *GRASP* deserve further consideration.

## Discussion

### Comparison with other studies

Through combined transcriptome and methylome analysis we have identified a panel of DNA methylation biomarkers that show a high frequency of methylation in colorectal cancers and adenomas; indeed a number of these were shown to be down-regulated in adenomas. In all, 23 of the 32 genes evaluated using qMSP in validation tissue samples were methylated in 50% or more of cancers (Figure 3). Using a variety of related approaches a number of groups have recently published candidate gene methylation biomarkers of colorectal cancer; McrBC fractionation/ microarray (CHARM) [23] combined gene expression and methylated DNA immunoprecipitation analysis [21], Infinium Human Methylation 27 K [6,18,20,24] and methylation capture sequencing [7]. We have combined analysis of gene expression with two novel methods of genome-wide DNA methylation characterisation. These different experimental approaches have led to the identification of candidate biomarker sets with substantial overlap and notably, many of our highly ranked markers have also been identified in other studies (Table 1) and are supported by DNA methylation microarray data from the TCGA (Additional file 1: Figure S1). For example, *IRF4* was among the candidate genes identified as methylated in CRC in three studies [18,20,24], including in adenomas [20] and TCGA data demonstrates strong differential methylation between cancer and normal tissues. The *SDC2* gene was ranked second by Simmer at al. [7] in a survey of genes methylated in CRC and its potential as a plasma biomarker was recently supported by Oh et al. [25]. For other genes, e.g. *FOXI2* and *SOX21*, their methylation in colorectal cancer has not previously been reported, but they are likewise supported by Infinium Human Methylation 27 K microarray data from the TCGA consortium. The breadth of concordance across multiple datasets, especially for biomarkers identified using different methods of genome-wide methylation analysis provides confidence in the potential of these genes as candidate biomarkers.

### Nature of the methylated genes

We have used Ingenuity Pathway Analysis to analyse the broader set of 72 genes directly selected using two genome-wide methods of DNA methylation analysis (combined lists from SuBLiME and Bisulfite-Tag analysis, Additional file 2: Tables S9 and S10). As has been observed in other similar studies [18,24] the set of genes methylated in CRC includes a high fraction (32/72) of nuclear proteins/transcription factors, particularly zinc finger proteins and homeobox-containing genes. There is also a high frequency of genes whose products localise to the plasma membrane (15 genes) or the extracellular space (9 genes). Within disease categories, the greatest enrichment is seen within “metastatic colorectal cancer” (p = 1.67E-5, Additional file 2: Table S10B). A number of the genes are functionally linked to development of the gastrointestinal or tract (p = 4.90E-9) and/or digestive system (p = 1.80E-8) (*DLX5*, *FOXF1*, *HOXA5*, *LHX6*, *NEUROD1*, *NKX2-2*, *NKX2-3*, *NKX2-6*, *ONECUT2*, *PDX1*, *PHC2*, *SALL1*), while 29 fall within the functional category Cellular development/ differentiation of cells (p = 2.69E-10), Additional file 2: Table S10A. The functional categories including development of endocrine glands (p = 1.1E-8), linking pancreas (p = 2.63E-6) and islet cells (p = 2.87E-6) also rank highly; it is notable that four of the methylated genes, *PDX1, NEUROD1, GDNF* and *NGN3* are critical in the development of pancreatic β cells [39,40]. 36 of the 72 genes are found within three regulatory networks, “Gene Expression, Cellular Development, Endocrine System Development and Function”, 17 genes, “Cellular Movement, Cardiovascular System Development and Function, Tissue Development”, 10 genes and “Cell Death and Survival, Lymphoid Tissue Structure and Development, Tissue Morphology”, 9 genes (Additional file 2: Table S10C).

Since regional gene silencing and DNA methylation or Long Range Epigenetic Silencing (LRES), defined as regions in the range from <1 Mbp to ~4 Mbp, has been observed in CRC [41] and other cancers [42,43] and one of our lead candidate genes, IKZF1 had been reported to be in an LRES region [33], we considered the location of genes we identified as methylated in CRC. Among the combined list of 74 Bisulfite-Tag and SuBLiME genes/regions, 23 genes were found within 3 Mb of at least one of the other genes - 8 pairs and one cluster each of 3 or 4 genes (Additional file 2: Table S11). Further inspection of the 1769 promoer proximal DMC identified in SuBLiME analysis [16] showed that all of these 10 regions harbored additional DMC (Addtional file 2: Table S11), with one region on Chromosome 19 including 29 genes with DMCs within 3 Mbp, many of them zinc finger genes, This indicates that genes methylated with high frequency in CRC are commonly found co-located with other methylated genes and conversely, that LRES of multiple regions may be common in CRC.

Comparison of genome-wide expression data with methylation data has demonstrated that most genes that become methylated in colorectal and other cancers are not expressed or expressed to a very low level in the normal tissue from which the cancer is derived [7,8], indicating that their methylation is not causative in silencing their expression or promoting cancer development. However, a proportion the methylated genes are active in normal tissue and it is among these that potential “drivers” are likely to be found. It is notable that among 23 genes we found to be methylated in at least 50% of neoplastic samples, 8 were initially identified among genes whose expression was down-regulated in colorectal neoplasia (*COL4A1*, ***COL4A2***, ***SDC2***, ***FOXF1***, ***MAFB***, ***EFEMP1***, ***ZSCAN18*** and *EPB4IL3*, Figure 3 and Table 1); the six highlighted in bold are also among the subset with significant down-regulation in adenomas (Additional file 2: Table S1).

### Potential for development of diagnostic assays

Colorectal cancer diagnostic assays of methylated sequences of either the *SEPT9* or *VIM* genes are now commercially available for detection in plasma and stool samples respectively and other genes such as *THBSI*[19] and *SDC2*[25] are also being evaluated for plasma-based diagnosis. While both *SEPT9* and *VIM* become methylated in a high fraction of colorectal cancers and adenomas, a recent comparison in a large set of colorectal tissue samples with other candidate methylation markers demonstrated the potential of additional markers to increase detection sensitivity [44]. Likewise, our comparison of the level of methylation of individual genes in different cancers (Additional file 3: Figure S3) supports the potential of multi-marker panels to provide increased sensitivity for detection in tissue samples, which is likely to be reflected in blood or stool-based assays.

For development of diagnostic assays for early detection of CRC, detection of methylated DNA biomarkers in both blood (plasma) and in fecal samples are being pursued. Different criteria need to be applied for marker selection in each case since the conditions of their release into these biological samples, their stability, their abundance and the technical challenges for detection remain to be determined. For detection of methylated, cancer-derived, DNA in feces it is preferable that there is minimal methylation in surrounding non-neoplastic colon tissue. Among the genes characterised in detail, six genes, *SOX21*, *SLC6A15*, *NPY*, *GRASP*, *ST8SIA1* and *ZSCAN18*, as well as *SEPT9*, were methylated in >50% of CRC samples and were not methylated to a level of >10% in any of the matched non-neoplastic samples analysed. It is also possible that methylation in normal colorectal tissue from subjects with neoplasia might arise in response to that neoplasia, or that adenomas and tumors arise within fields of histologically normal tissue that harbor epigenetic changes. In such circumstances, markers showing a neoplasia-related “field effect” could be investigated further as biomarkers of risk of cancer or as potentially more sensitive markers for identification of cancer-related DNA in fecal samples. For use as biomarkers for detection of cancer DNA in blood, either plasma or serum, it is important that the background in the blood of normal subjects is minimal. While the source of free DNA in plasma or serum of normal subjects is not well understood, a likely major source either from in vivo cell lysis or lysis during sample handling is white blood cells themselves. Using a cut-off of 0.1% methylation in wbc DNA, 15 of the 23 genes that showed methylation in at least 50% of cancers and adenomas, and particularly 11 genes methylated in at least 70% of neoplastic samples (*BCAT1*, *COL4A2*, *DLX5*, *FGF5*, *FOXF1*, *FOXI2*, *GRASP*, *IKZF1*, *IRF4*, *SDC2* and *SOX21*) show potential for evaluation as biomarkers for CRC detection in blood. Several of these show significant levels of methylation in normal colon tissue and so would not be suitable as biomarkers for use in feces. The lack of methylation detected in wbc DNA for some of these genes, notably *IKZF1*, *IRF4*, *BCAT1*, and very low levels for others, e.g. *COL4A2*, *DLX5*, *SOX21* and *GRASP*, suggest that these represent good candidates for further development, either as individual biomarkers or as components of panels that might provide increased sensitivity and specificity of early detection of CRC.

## Conclusions

This study has characterised a panel of 23 genes that show elevated DNA methylation in at least 50% of CRC tissue relative to control non-neoplastic tissue. Six of these genes (*SOX21*, *SLC6A15*, *NPY*, *GRASP*, *ST8SIA1* and *ZSCAN18)* show a very low level and frequency of methylation in non-neoplastic colorectal tissue and are candidate biomarkers for stool-based assays. 11 genes (*BCAT1*, COL4A2, *DLX5*, *FGF5*, *FOXF1*, *FOXI2*, *GRASP*, *IKZF1*, *IRF4*, *SDC2* and *SOX21*) show very low methylation levels in wbc DNA from healthy subjects and hence are suitable for further evaluation as blood-based CRC diagnostic biomarkers.

## Abbreviations

cDNA: Complementary DNA; CIMP: CpG Island Methylator Phenotype; CRC: Colorectal cancer; d-Aza: 5-Aza-2′-deoxycytidine; DMC: Differentially methylated CpG site; IPA: Ingenuity Pathway Analysis; LRES: Long Range Epigenetic Silencing; MSI: Microsatellite-instable; qMSP: Quantitative methylation specific PCR; qPCR: Quantitative PCR; SuBLiME: Streptavidin bisulfite ligand methylation enrichment; TSA: Trichostatin A; wbc: White blood cell.

## Competing interests

CSIRO has received partial funding from Clinical Genomics Pty Ltd for the work described in this manuscript. IB, AM, MLT, RTB SKP and LCL are employees of Clinical Genomics Pty Ltd, who have partly funded this work. GPY is a consultant to Clinical Genomics Pty Ltd. LCL, RD, GPY, PLM, SKP, TJL, JPR, HRD, SMM, KRD & MJB are inventors on one or more patent applications covering candidate biomarkers described in this paper. The patents are assigned jointly to CSIRO and Clinical Genomics Pty Ltd.

## Authors’ contributions

SMM contributed to experimental planning, design and development of PCR assays and amplicons for sequencing library preparation, co-ordination of data and paper preparation. JPR provided SuBLiME experimental data and analysis, analysis of amplicon sequence data and TCGA microarray data. HRD developed the Bisulfite-tag technique and applied it to the clinical specimens. TH conducted qMSP assays on clinical samples, optimised amplicon preparation for amplicon sequencing. GSB contributed to the experimental development of Bisulfite-tag, carried out gene expression and Bisulfite-tag microarray experiments and contributed to PCR amplicon design. NFWS conducted bioinformatic analyses of gene expression data. KRD contributed to bioinformatic analyses of Bisulfite-tag and gene expression microarray array data. MJB contributed to bioinformatic analyses of Bisulfite-tag array data. RD contributed to bioinformatic analyses of gene expression data. IB contributed to bioinformatic analysis of amplicon sequencing data. KNR provided ongoing input into experimental design and data interpretation. AM, MLT and RTB contributed to the design, optimisation and conduct of qMSP assays. DAW organised access, QC and provision of clinical samples and associated data. GPY contributed to overall project design, clinical interpretation, sample choice and provision. TJL participated in the design of the study and data interpretation. SKP contributed to the ongoing experimental design and data interpretation of the study and oversaw and co-ordinated sections of the qMSP work. LCL contributed to conception of the study, and provided ongoing input into data interpretation and project directions. PLM contributed to conception and development of the project, experimental design, data interpretation and manuscript preparation. All authors read and approved the final version of the manuscript.

## Pre-publication history

The pre-publication history for this paper can be accessed here:

http://www.biomedcentral.com/1471-2407/14/54/prepub

## Supplementary Material

Additional file 1 **Mitchell et al., A panel of genes methylated with high frequency in colorectal cancer. Figure S1.** Boxplots of methylation TCGA consortium data. The fraction of methylated cytosine (beta value) at CpG sites is shown for CRC (red) and normal colorectal tissue (blue). **Figure S2.** Heatmaps of MSP data. Upper heatmap includes all tumors and markers. The lower panels show heatmaps for 7 markers (75 tumors) and expanded set of 12 markers (20 tumors). The colour scale is a palette of nine colours from yellow to green to blue and is representative of the methylation rate, with a bluer colour denoting hypermethylation, as detected by the assay. The data presented in the heatmaps was log2 normalised (with 1% methylation added first). The colour in the vertical bars on the left denote the stage of the tumours (A, B, C, D), with a redder colour, a later stage cancer and a yellow colour, an adenoma (Ad). The colours for each stage or adenoma are presented in the legend on the heatmap. Areas of white in the upper heatmap denote missing data. Click here for file

Additional file 2: Table S1 Genes downregulated in colorectal neoplasia. **Table S2.** Reactivation of gene expression in cell lines using 5′ 2-deoxycytidine and/or trichostatin. **Table S3.** Amplicons and primer pairs for Roche 454 amplicon sequencing. **Table S4.** Primers, probes and amplification conditions for methylation specific PCRs. **Table S5.** Differentially methylated genes and regions as determined by bisulfite-tag. **Table S6.** Differentially methylated genes and regions as determined by SuBLiME. **Table S7.** Methylation levels across amplicons as determined by Roche 454 multiplexed amplicon sequencing. **Table S8.** Methylation frequency of candidate genes as determined by qMSP. **Table S9.** Ingenuity Pathway Analysis: cellular location and functional grouping of the gene products. **Table S10A.** Ingenuity Pathway Analysis: top biological functions. **Table S10B.** Ingenuity Pathway Analysis: top disease functions. **Table S10C.** Ingenuity Pathway Analysis: top gene networks (genes from list highlighted in red). **Table S11.** Combined top genes and regions from bisulfite-tag and SuBLiME analysis. Click here for file

Additional file 3: Figure S3 Pairs plot comparing methylation levels of different genes. Log2transformed methylation levels are plotted pairwise in separate panels for twelve genes (lower left panels). Cancer samples are shown as red dots and adenoma samples as purple triangles.Within each pairs plot the grey diagonal line represents equivalent levels of methylation. Pearson correlation coefficients for each gene pair are shown in the upper right half of the figure, together with the number of contributing pairs in brackets. Click here for file
