# Supplementary material for: A panel of genes methylated with high frequency in colorectal cancer
Source: BMC Cancer. 2014 Jan 31;14:54. doi: 10.1186/1471-2407-14-54 (PMC3924905; doi:10.1186/1471-2407-14-54)
Supplement: Additional file 1 — Mitchell et al., A panel of genes methylated with high frequency in colorectal cancer. Figure S1. Boxplots of methylation TCGA consortium data. The fraction of methylated cytosine (beta value) at CpG sites is shown for CRC (red) and normal colorectal tissue (blue). Figure S2. Heatmaps of MSP data. Upper heatmap includes all tumors and markers. The lower panels show heatmaps for 7 markers (75 tumors) and expanded set of 12 markers (20 tumors). The colour scale is a palette of nine colours from yellow to green to blue and is representative of the methylation rate, with a bluer colour denoting hypermethylation, as detected by the assay. The data presented in the heatmaps was log2 normalised (with 1% methylation added first). The colour in the vertical bars on the left denote the stage of the tumours (A, B, C, D), with a redder colour, a later stage cancer and a yellow colour, an adenoma (Ad). The colours for each stage or adenoma are presented in the legend on the heatmap. Areas of white in the upper heatmap denote missing data. [file 1471-2407-14-54-S1.pdf]

Additional File 1:

Mitchell et al., A panel of genes methylated with high frequency in colorectal cancer

1. Deoxy - Azacytidine and Trichostatin mediated gene activation

HCT116 and HT29 cells were cultured in McCoy's medium plus 10% Fetal Bovine Serum (FBS) and SW480 cells in a 50:50 mix of D-MEM and F12 medium, supplemented with 10% FBS. LIM1215 cells were cultured in HEPES buffered RPMI1640 medium supplemented with 2mM L-Glutamine+25mM, 0.6μg/ml Insulin, 1μg/ml Hydrocortisone, 10 μM 1-Thioglycerol and 10% FBS.

Cell lines were treated with 5-aza 2deoxycytine (d-Aza) alone, or in the presence of trichostatin (TSA), since TSA in combination with d-Aza frequently gives improved activation of expression of DNA-methylated genes compared with d-Aza alone. This is presumably because some demethylated genes remain partially repressed because of histone modifications, e.g. [1]. Cells were treated for 48 hr at the concentrations shown in Additional file 2, Table S1 and allowed to grow in the absence of treatment for a further 24 hr before they were harvested and DNA and RNA isolated. The extent of demethylation induced by the treatments was determined using an End-specific PCR assay for the level of unmethylated Alu repeat sequence [2]. The leukemic cell line, K562, that is known to be highly hypomethylated was used for reference. Input DNA levels were determined using a reference rRNA gene assay in the same reaction. For three of the cell lines improved demethylation was seen in the presence of both d-Aza and TSA. The samples used for exon array gene expression analysis, their treatment conditions and their level of demethylation relative to the K562 line, are shown in Table S1 (Additional file 2). The cell lines HCT116, HT29 and SW480 exhibited high levels of demethylation in comparison with LIM1215 where toxicity limited the levels of d-Aza and TSA that were used for treatment.

From control and treated cells, cDNA was prepared and hybridised to Affymetrix Exon 1.0ST microarrays. Probesets with differential expression (treated - control) within cell lines were identified using *limma*. Probes associated with gene names within the list of 429 downregulated genes (Additional file 2, Table S1) were ordered by log(fold change) within each cell line. The maximum logFC for probesets in each cell line was taken and the mean logFC across the four cell lines determined. Data for the set of genes chosen for further analysis (main paper, Table 1) is shown in Table S2 (Additional File 2). Consistent with the level of activation of repeat sequences, genes activation was most frequent in HCT116 and least frequent in LIM1215 cells. Genes with a LogFC of <1 (ie 2-fold change in gene expression) were chosen for further analysis prior to obtaining the d- Aza/TSA data.

2. Bisulfite-tagging

Data from microarrays for (a) the eight individual cancer and normal pairs (b) from the analysis of pooled DNA from the cancer and normal tissues and (c) the dye-swap hybridisation with pooled DNAs were combined as described below to identify the most

differentially methylated genes. Differential methylation was assessed via a difference-difference score, DD. This was defined for each probe as:

DD = (Y.meth.tumour - Y.unmeth.tumour) - (Y.meth.normal - Y.unmeth.normal),

where each Y value is the base-2 logarithm of the raw probe response value for the given combination of methylation status (meth/unmeth) and disease status (tumour/normal). Larger values of DD imply methylation is increased in the tumour samples relative to normal samples. For each tiled region probes were ranked in order of highest DD, as DD1, DD2, DD3 and so forth.

Two gene lists were created, one that was based on the differential methylation for the two most differential probes in a tiled region (DD2) and one that was based on the top four differential probes (DD4). For the DD2 list, we discarded probes where the probe quality as scored by the Nimblegen microarray software, was beyond the threshold of 1.5. This threshold accepts about 17% of all probes and minimises the potential for “noise” when only two probes are considered. For each tiled region, the DD2 values for the eight individual samples were combined into a median. Then an overall DD2 value across all samples obtained from the average of three values: this median, and the two pooled sample values, forward and dye-swap. All tiled regions were then ranked by this score, with the top 30 returned.

For the DD4 list, where a greater number of probes per tiled region were considered, a quality score threshold of 4 (that accepts about 40% of probes) was used. The overall DD4 value for each tiled region was determined as for DD2, except using the individual DD4 values in each case.

Table S5 (Additional file 2) shows a list of 44 genes shown to be differentially methylated between cancer and normal DNAs; based on either the level of the differential signal from the DD2 or DD4 analysis. The list is ordered by the difference based on 4 probes, and then 2 probes. The top 4 ranked probes (and 9 in all) appear in the top 30 gene list derived by both methods. The table lists HGNC gene symbols, where available, in the first column, followed by hg19 coordinates for the gene loci and then of the tiled regions on the Nimblegen arrays showing differential methylation. The last three columns show the DD2 and DD4 scores, as well as the rank among 1802 genes identified as differentially methylated in SuBLiME data (see below).

3. SuBLiME methylation analysis

Table S6 (Additional file 2) provides an alphabetical listing of genes and SuBLiME data for genes analysed in this manuscript. A full list of differentially-methylated genes has been published previously [3].***4. Selection of genes***

An initial set of genes was chosen from among the 429 that we had previously shown to be down-regulated in a high fraction of CRC specimens (left hand Venn diagram below). Some initial genes (e.g. *CFD* and *MT1M*) were chosen based only on their level of down-regulation, but were not later found to be supported by genome-wide methylation data or to be reactivated by d-Aza/TSA treatment. Five genes, *COL1A2*, *EDIL3*, *FBN1*, *MEIS1* and *SDC2*, were reactivated by d-Aza/TSA treatment and also supported by both SuBLiME and Bis-Tag data. Methylation of some genes, e.g. *ADAMTS1*, *EFEMP1*, *COL4A2* and *EPB4IL3*,

was also supported by literature data (Table 1). Independently of the original set of downregulated genes we further evaluated the the top candidate genes from the genome-wide Bis-tag and SuBLiME data. The Venn diagram to the right shows the additional genes selected and whether they were further supported by either TCGA data or other literature (Table 1). Within the candidate gene groups, a number were not taken further because of the limited number of CpG sites or because it did not prove possible to develop clean assays for them.

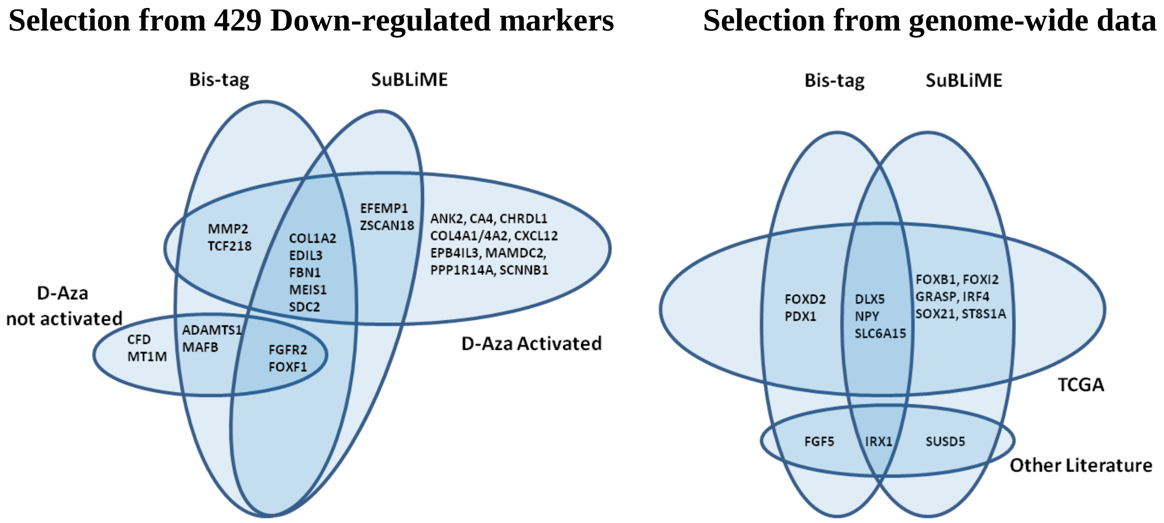

5. Multiplexed Bisulfite sequencing

Primer pairs and reaction conditions for individual promoter regions are shown in Table S3 (Additional file 2), along with chromosomal co-ordinates. Amplicons were prepared from bisulfite treated DNA of 10 colorectal cancer specimens, their matched normal tissue and normal blood DNA. Amplification was done using Promega GoTaq master mix (without SYBR Green I), 4mM $MgCl_2$ and with primers at 200nM and 10ng of input DNA. Cycling conditions were 95°C, 2 min (1 cycle), followed by 50 cycles of 95°C 15 sec, N°C 30 sec; 72°C 30 sec. The annealing temperature, N°C ,for each amplicon is shown in Table S3 (Additional file 2). For some amplicons (Table S3) an additional 200µM of dATP and dTTP was added to enable comparable amplification of both methylated and unmethylated DNA sequences. Amplified bands of DNA were gel purified and equivalent amounts of the separate amplicons derived from each DNA sample were pooled and ligated with linkers for sequencing on the Roche 454 Titanium FLX system. Samples from individual patient's cancer or normal DNA and the blood DNA sample were separately ligated with bar coded "MID" linkers (Roche Cat No 05619211001) so that sequence reads could later be assigned to individual samples for sequence alignment and scoring. Libraries were prepared following protocols provided with the Roche Library preparation kit and reagents and sequenced on two halves of a flow cell; one half contained all the cancer samples and one the equivalently bar-coded normal samples. The bisulfite sequencing reads were segregated to individual samples with a Python script using the bar-code sequences and aligned with the bisulfite converted sequence of each amplicon using SHRiMP V2.04. After alignment, the output files were

parsed and the fraction of cytosines at each potential CpG methylation site was determined for each sample using a custom R script.

Table S7 (Additional file 2) shows for each amplicon the mean level of methylation at CpG sites across the amplicon for (Column E) all cancers combined, (column F) all matched normal samples combined, (Column H) wbc DNA, (Column I) a 50:50 mix of fully methylated and wbc DNAs and (Columns J – AC) all ten individual cancers. Columns C and D shown how many of the ten cancers showed high level methylation (>50%) or partial methylation (20-50%). The 10 pairs of cancer and normal were compared using a paired Wilcoxon signed-rank test, a non-parametric equivalent of the paired samples t-test. The alternate hypothesis was that cancer samples had greater methylation than normal samples (a one-sided test).

6. Methylation-specific PCR primers and amplification conditions.

Primers for methylation specific PCR, used for quantification of DNA methylation levels in tissue DNA samples, are shown in Table S4 (Additional file 2), along with reaction buffers and amplification conditions. Where used, Taqman probes are also shown; otherwise detection was performed with SybRGreen. Also shown are primers and reaction conditions for the *CFF* assay used to quantify input DNA levels.

7. Comparison with TCGA data

Illumina 27k Methylation Bead Chip level 1 data for 165 colorectal primary tumours and 37 non-neoplastic colorectal tissues [34] was downloaded from the TCGA consortium website and a custom R script was used to parse the data into a MethylLumiM object [45]. From here, the array data was corrected for colour bias and normalised using shift and scaling normalisation (ssn). Probes with a call rate of >=90% had β and M values extracted. The β values were used to plot cancer and normal values in box plots. These were plotted for genes of interest and used to identify those for which there was good support in this independent cohort. Genes in Table 1 for which TCGA data demonstrated cancer-specific methylation are identified in Table 1. Boxplots for 15 genes are shown in Figure S1.

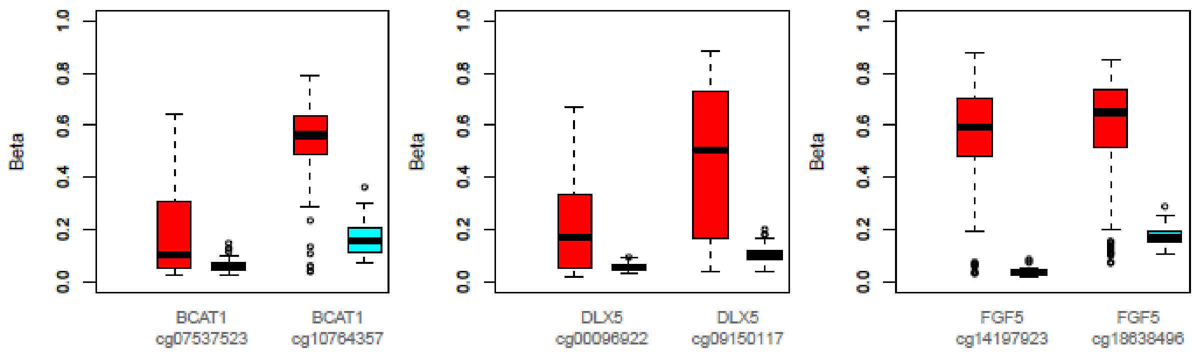

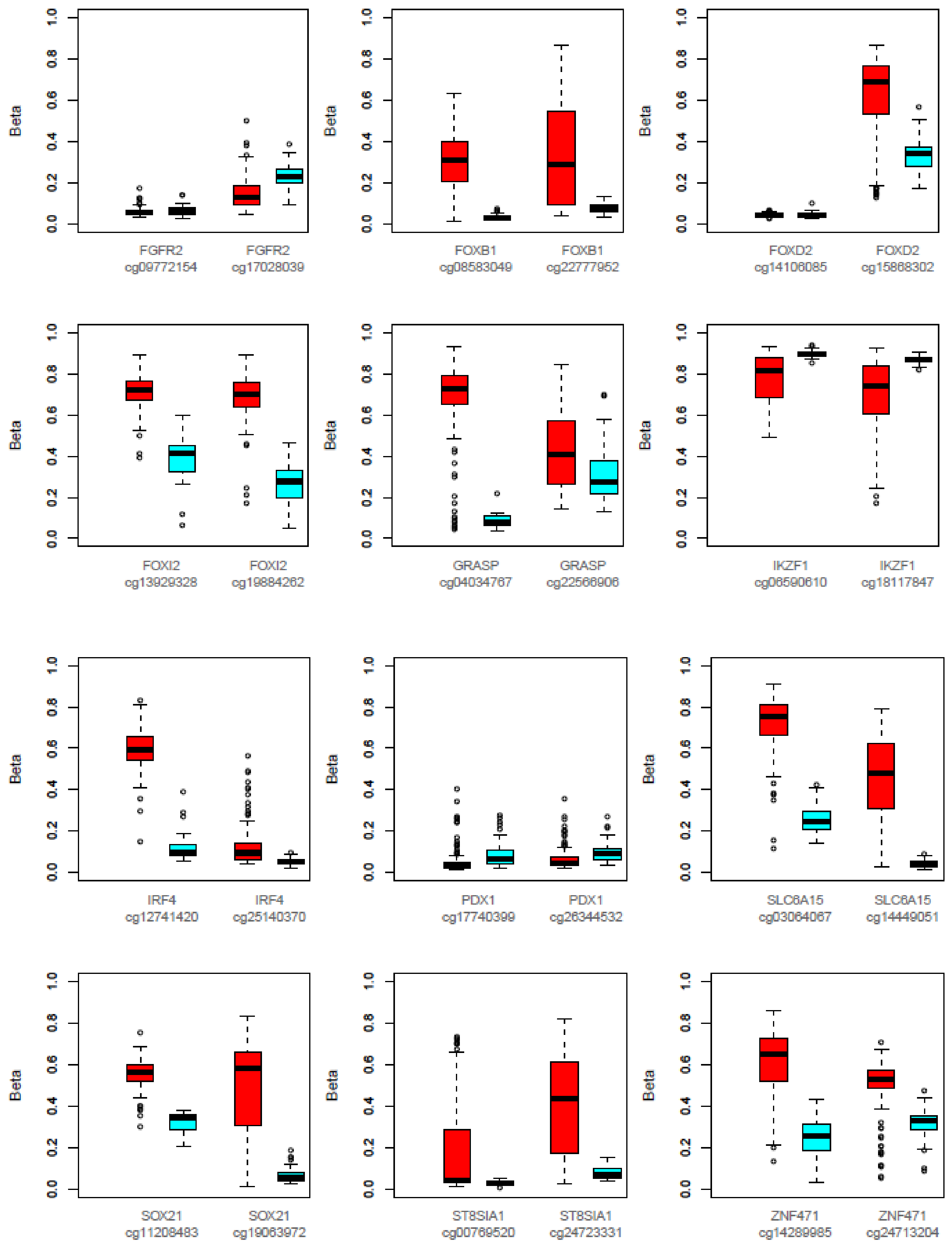

Figure S1. Boxplots of methylation TCGA consortium data. The fraction of methylated cytosine (beta value) at CpG sites is shown for CRC (red) and normal colorectal tissue (blue)

7. MSP assays on DNA from cancer, adenoma and normal issue

For each gene tested, Table S8 (Additional file 2) shows the percentage of cancer, adenoma or normal tissue DNA samples that were methylated to a level of >10%. The table also

includes the number of samples of each type assayed for each gene. The final column shows the difference in detection cycle (Ct) between fully methylated DNA and wbc DNA. Data is presented pictorially in Figure 3.

8. Relative methylation of different genes in neoplastic tissue DNA

qMSP-derived values for % methylation of genes in individual neoplastic tissue DNA samples was used to cluster be tumor sample and marker gene. The heat maps, Figure S2, incorporates data for 7 markers from 75 tumours and for a subset of 20 tumors with data for an expanded set of 12 markers.

Some of the MSP data had an estimated methylation rate substantially above 100%. This may be caused by inaccuracies in the method, or phenomena in the tumour such as gene duplications or deletions that can lead to different copy numbers for the target and reference genes. To reduce the visual impact of these on the heatmap, the estimated percentage methylation data had 1% added and was log2 normalised before heatmap construction.

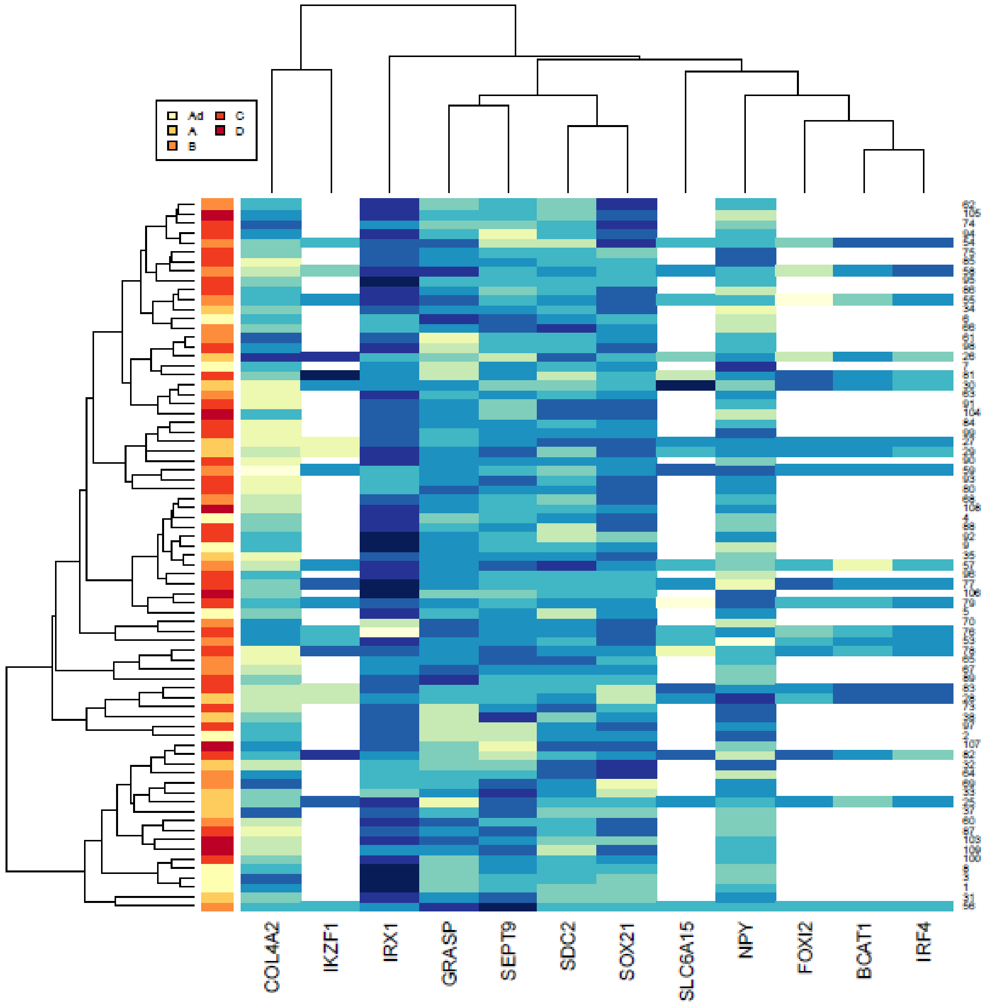

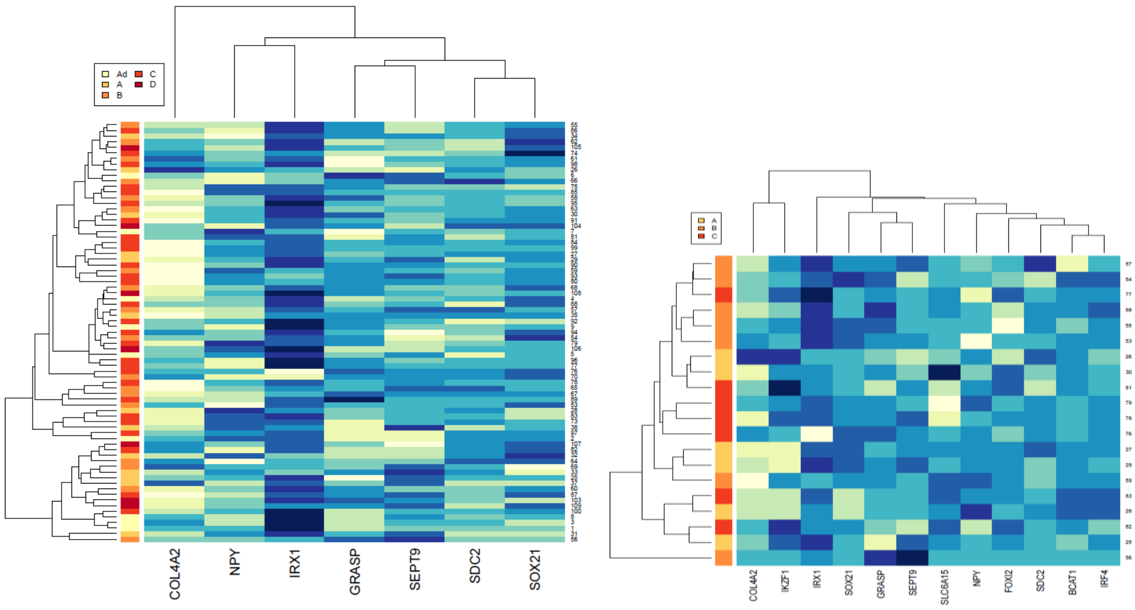

Figure S2. Heatmaps of MSP data. Upper heatmap includes all tumors and markers. The lower panels show heatmaps for 7 markers (75 tumors) and expanded set of 12 markers (20 tumors). The colour scale is a palette of nine colours from yellow to green to blue and is representative of the methylation rate, with a bluer colour denoting hypermethylation, as detected by the assay. The data presented in the heatmaps was log2 normalised (with 1% methylation added first). The colour in the vertical bars on the left denote the stage of the tumours (A, B, C, D), with a redder colour, a later stage cancer and a yellow colour, an adenoma (Ad). The colours for each stage or adenoma are presented in the legend on the heatmap. Areas of white in the upper heatmap denote missing data.

9. Ingenuity Pathway Analysis

A list of 72 methylated genes was formed by combining the differentially-methylated gene lists from Bisulfite-tag and SuBLiME analyses (Tables S4 and S5, Additional file 2). This gene name list of the top 72 markers was uploaded into Ingenuity Pathway Analysis. A core analysis was performed where only molecules and/or relationships were considered when the species was human and the confidence was high in either predicted or experimentally observed variables in the IPA data base. Table S9 (Additional file 2) shows the location and functional grouping of the gene products. Table S10 (Additional file 2) includes the top ranked biological process (Table S10A), disease processes (Table S10C) and regulatory networks (Table S10C).

Additional References

1. Gagnon JF, Bernard O, Villeneuve L, Têtu B, Guillemette C: **Irinotecan inactivation is modulated by epigenetic silencing of UGT1A1 in colon cancer.** Clin Cancer Res. 2006, 12(6):1850-8

2. Rand KN, Molloy PL: **Sensitive measurement of unmethylated repeat DNA sequences by end-specific PCR**. *Biotechniques* 2010, **49**(4):xiii-xvii.
3. Ross JP, Shaw JM, Molloy PL: **Identification of differentially methylated regions using streptavidin bisulfite ligand methylation enrichment (SuBLiME), a new method to enrich for methylated DNA prior to deep bisulfite genomic sequencing**. *Epigenetics* 2013, **8**(1):113-127.
4. Muzny DM, Bainbridge MN, Chang K, Dinh HH, Drummond JA, Fowler G, Kovar CL, Lewis LR, Morgan MB, Newsham IF *et al*: **Comprehensive molecular characterization of human colon and rectal cancer**. *Nature* 2012, **487**(7407):330-337.
5. Du P, Kibbe WA, Lin SM: **lumi: a pipeline for processing Illumina microarray**. *Bioinformatics* 2008, **24**(13):1547-1548.
