# Supplementary material for: A panel of genes methylated with high frequency in colorectal cancer
Source: BMC Cancer. 2014 Jan 31;14:54. doi: 10.1186/1471-2407-14-54 (PMC3924905; doi:10.1186/1471-2407-14-54)
Supplement: Additional file 3: Figure S3 — Pairs plot comparing methylation levels of different genes. Log2transformed methylation levels are plotted pairwise in separate panels for twelve genes (lower left panels). Cancer samples are shown as red dots and adenoma samples as purple triangles.Within each pairs plot the grey diagonal line represents equivalent levels of methylation. Pearson correlation coefficients for each gene pair are shown in the upper right half of the figure, together with the number of contributing pairs in brackets. [file 1471-2407-14-54-S3.pdf]

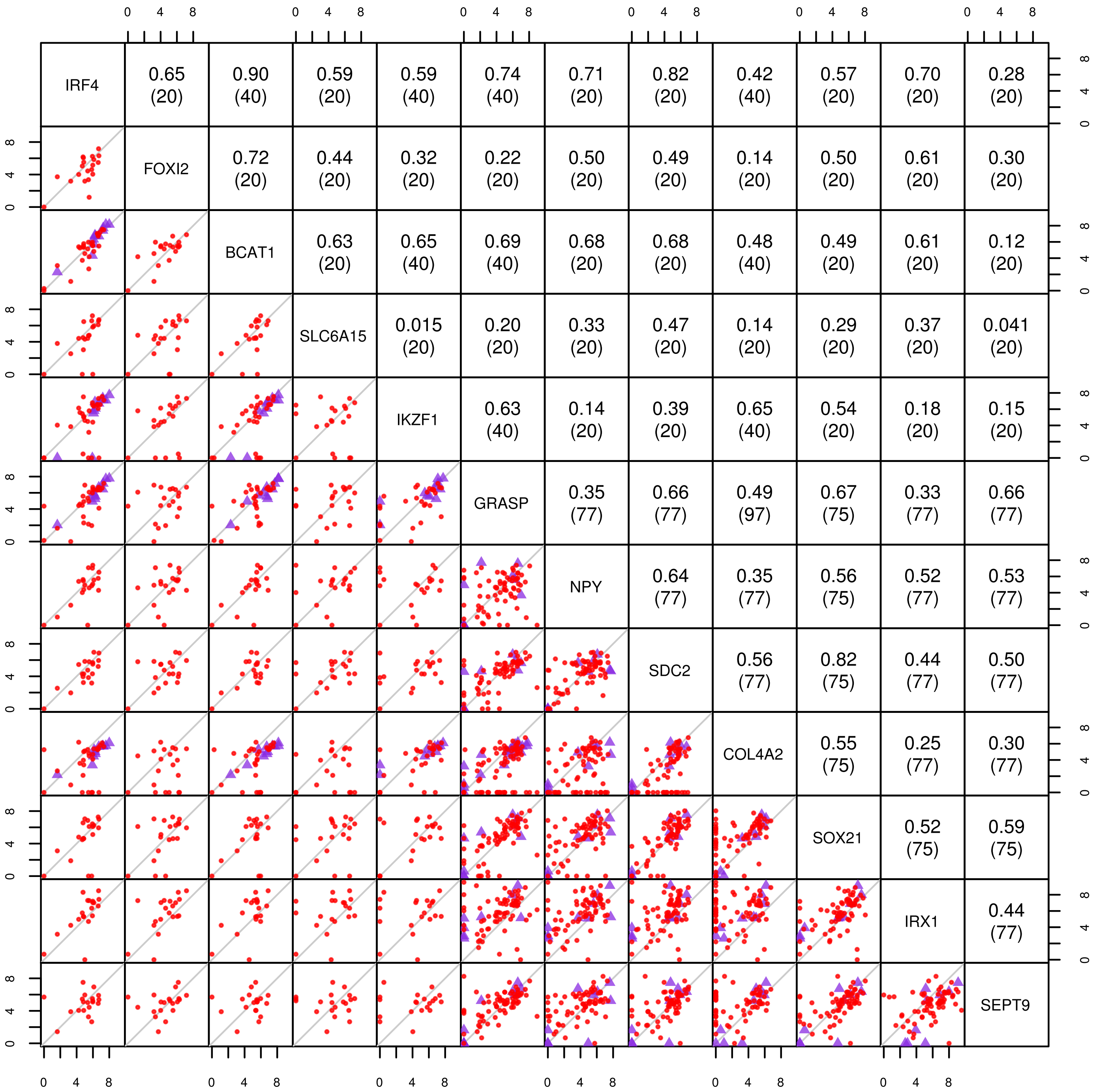

Figure S3. Pairs plot comparing methylation levels of different genes.

Log2transformed methylation levels are plotted pairwise in separate panels for twelve genes (lower left panels). Cancer samples are shown as red dots and adenoma samples as purple triangles. Within each pairs plot the grey diagonal line represents equivalent levels of methylation. Pearson correlation coefficients for each gene pair are shown in the upper right half of the figure, together with the number of contributing pairs in brackets.
